# A rare case of malignant triton tumor in the cerebellopontine angle

**DOI:** 10.1186/1746-1596-7-43

**Published:** 2012-04-19

**Authors:** Li Gong, Xiao-Yan Liu, Wen-Dong Zhang, Xiu-Juan Han, Li Yao, Shao-Jun Zhu, Miao Lan, Yan-Hong Li, Wei Zhang

**Affiliations:** 1 Helmholtz Sino-German Laboratory for Cancer Research, Department of Pathology, Tangdu Hospital, Fourth Military Medical University, Xi’an, 710038, People’s Republic of China; 2 Department of Gynaecology and Obstetrics, Tangdu Hospital, Fourth Military Medical University, Xi’an, 710038, People’s Republic of China

**Keywords:** Malignant triton tumor, Malignant peripheral nerve sheath tumor, S-100 protein, Myoglobin

## Abstract

**Abstract:**

Malignant triton tumor (MTT) is defined as malignant peripheral nerve sheath tumor with rhabdomyoblastic differentiation. Intracranial MTT is extremely rare, and only four cases have been reported in the literature. Here, we report a case of MTT occurring in the cerebellopontine angle, and describe its histopathological characteristics, immunohistochemical features, and prognosis.

**Virtual slides:**

The virtual slide(s) for this article can be found here: http://www.diagnosticpathology.diagnomx.eu/vs/1336227313684480

## Introduction

 Malignant peripheral nerve sheath tumor (MPNST) is an uncommon type of sarcoma that arises from peripheral nerve sheaths. The origin of this tumor is thought to be Schwann cells or pluripotent cells of the neural crest. About 50 % of MPNSTs are known to develop into cases of neurofibromatosis type 1 (von Recklinghausen’s disease, NF-1) [1]. Malignant triton tumor (MTT), which is a subtype of MPNST in which malignant Schwann cells coexist with malignant rhabdomyoblasts, was firstly reported by Masson et al. in 1932 [2]. MTT is extremely rare, and accounts for less than 5 % of all MPNSTs [3]. The average age of patients with MTT is 31.7 years, and the morbidity is approximately equal in males and females [4]. MTT usually occurs in the head, neck, and trunk [3,5,6]. Other rare positions, such as the retroperitoneal space, parapharyngeal space, lumbar spine, cervical spine, intracardiac, and nose, have also been reported in the literature [7-12]. Intracranial MTT is very rare, and only four cases have been described [13-16]. Here, we describe a case of MTT occurring in the cerebellopontine angle.

## Case presentation

**Figure 1:**
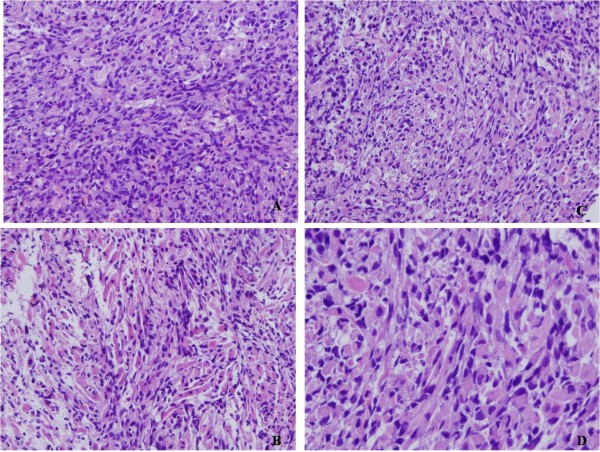
**Microscopically, the tumor was composed of spindle cells with wavy nucleus and scattered or focal large cells which were rounded or elongated with abundant deep eosinophilic cytoplasm.**Cross striations were found in few of them. Mitotic figures were scarce in the cellular areas. A, ×200; B, ×200; C, ×200; D, ×400.

**Figure 2:**
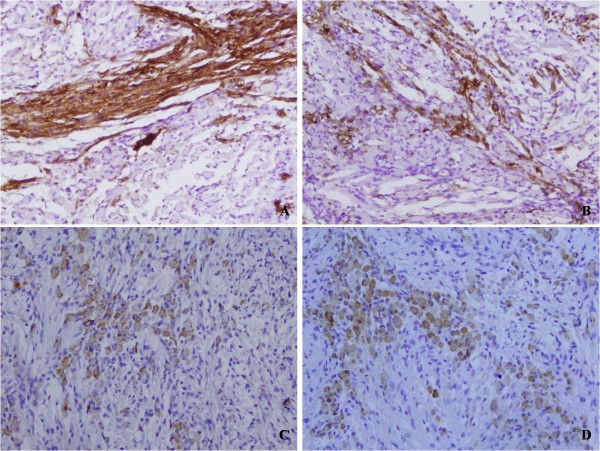
Immunohistochemically, the spindle cells were positive for S-100 protein (A, ×200; B, ×200 ), and the large cells were positive for desmin (C, ×200) and myoglobin (D, ×200).

 A 55-year-old female was referred to our hospital because the left angle of her mouth had been slanted and numb for six months, and she had experienced auditory dysesthesia for one month. Magnetic resonance imaging (MRI) revealed that there was a small mass in the left cerebellopontine angle. During the operation, the small mass, which was dark red and soft in texture, was found at the side of brainstem, and closely conglutinated with the surrounding cranial nerves. The resected mass measured 1.2×1.0×0.8 cm, and the cut surface was dark red. Microscopically, the tumor was composed of spindle cells with wavy nuclei, and scattered or focal large cells that were rounded or elongated with abundant and deep eosinophilic cytoplasm. Cross striations were found in a few cells. Mitotic figures were scarce (Figure 1). Immunohistochemically, the spindle cells showed focal positivity for S-100 protein and nerve fibers (NFs), and the large cells were positive for desmin and myoglobin (Figure 2). Both cell types showed negative staining for glial fibrillary acidic protein (GFAP), neuron specific enolase (NSE), HMB45, melanoma-pan, cytokeratin (CK), epithelial membrane antigen (EMA), alpha-fetoprotein (AFP), CD30, human chorion gonadotropin (HCG), SM-action, CD68, CD163, and CD34. Thus, we diagnosed this case as malignant peripheral nerve sheath tumor with rhabdomyoblastic differentiation, namely MTT.

The diagnosis of MTT was mainly based on histopathological characteristics and immunohistochemical features. Woodruff et al. [17] provided three diagnostic criteria for MTT: 1) the tumor originates from the surrounding nerve or develops into NF-1; 2) spindle cells are the main cell type in MPNST; 3) there are true neoplastic rhabdomyoblasts, but they are not due to involvement or metastasis of rhabdomyosarcoma in other positions. In our case, the characteristics, including imageology and histopathology, were in accordance with the above three criteria. Moreover, no masses were found in other positions. Hence we diagnosed it was a case of primary MTT in the cerebellopontine angle.

MTT should be differentiated from the following malignant tumors because of its complex and diverse histomorphology: 1) MPNST does not harbor rhabdomyoblastic differentiation, although the tumor cells are spindle-shaped. In addition, the tumor cells are negative for skeletal muscle proteins, such as desmin and myoglobin, and are positive for S-100 protein; 2) In malignant fibrous histiocytoma, the tumor is composed of spindle cells and giant tumor cells. However, the tumor cells do not express S-100 protein, desmin, or myoglobin, but do express AACT and CD68; 3) In leiomyosarcoma, the pattern of growth is predominantly fascicular, with the tumor bundles intersecting each other at wide angles. Merging of the tumor cells with blood vessel walls is an important diagnostic clue. The individual cells have elongated, blunt-ended nuclei and acidophilic fibrillary cytoplasm; features that are also apparent in cytologic preparations. Cytoplasmic vacuoles, which are located at both ends of the nucleus, can sometimes be indented as another diagnostic clue. Immunohistochemically, the tumor cells are positive for SM-actin and desmin, but negative for myoglobin and S-100 protein; 4) Rhabdomyosarcoma, especially embryonal rhabdomyosarcoma, is composed of undifferentiated rounded and spindle cells. Interestingly, well-differentiated rhabdomyoblasts have a deeply acidophilic cytoplasm, and are thus sometimes mis-diagnosed as MTT. However, rhabdomyosarcoma has a different composition than MPNST, and the average age of patients with this disease is younger than those with MTT. Moreover, a feature of diagnostic value is the presence of highly cellular areas that usually surround blood vessels, alternating with parvicellular regions that have abundant mucoid intercellular material. Immunohistochemically, the tumor cells show negative reactivity for S-100.

Like most soft-tissue sarcomas, MTTs are traditionally insensitive to chemotherapy and radiotherapy. However, several recent reports have suggested that neoadjuvant therapy and adjuvant chemotherapy can eradicate micrometastasis. Integrated positron emission tomography and computed tomography have been used to assess remission and response to therapy [18]. Thus, multidisciplinary treatments, including complete resection, are required for patients with MTT. McConnell et al. reviewed 124 cases of MTT reported in the literature, and concluded that complete surgical resection and adjuvant radiotherapy should be the cornerstone of MTT treatment [19].

The prognosis of MTT is very poor, with a 5-year survival rate of only 14 % and a median time to death of 13 months. The overall local recurrence/progression rate is 50 %, and the median time to recurrence/progression is six months [19]. In our study, the patient was discharged on post-operative day 12 with an uneventful recovery. Currently, the patient is alive with no evidence of disease after a 5-month follow-up.

There is limited information in the literature regarding cytogenetic analyses of MTT, and only eight cases of MTT have been previously karyotyped. Most of them showed rather complex cytogenetic deviations, although some genetic aberrations appear to be common [20-25]. Koutsimpelas et al. firstly analyzed the genomic imbalance of a case of MTT using comparative genomic hybridization (CGH) [26]. The results demonstrated the involvement of oncogenes located at chromosomes 1, 16, 17, 19, and 22. These studies will be important for the understanding of the pathophysiology of MTT.

## Conclusions

In conclusion, intracranial MTT is extremely rare. Its diagnosis must be based upon histopathological characteristics and immunohistochemical features.

## Consent

Written informed consent was obtained from the patient for publication of this Case Report and any accompanying images. A copy of the written consent is available for review by the Editor-in-Chief of this journal.

## Abbreviations

MTT = Malignant triton tumor; MPNST = Malignant peripheral nerve sheath tumor; MRI = Magnetic resonance imaging; NF = Nerve fiber; GFAP = Glial fibrillary acidic protein; NSE = Neuron specific enolase; CK = Cytokeratin; EMA = Epithelial membrane antigen; AFP = Alpha-fetoprotein; HCG = Human chorion gonadotropin; CGH = Comparative genomic hybridization.

## Competing interest

The authors declare that they have no competing interest.

## Authors’ contributions

LG selected the research topic, participated in the study, and wrote the manuscript. WDZ participated in writing the manuscript. YHL and WZ provided grant support. XYL and LY participated in the study. SJZ and XJH conducted the pathological examination. ML provided the technique support. All authors have read and approved the final manuscript.
